# Chondroitin Sulfate Proteoglycan *CSPG4* as a Novel Hypoxia-Sensitive Marker in Pancreatic Tumors

**DOI:** 10.1371/journal.pone.0100178

**Published:** 2014-06-16

**Authors:** Shereen Keleg, Alexandr Titov, Anette Heller, Thomas Giese, Christine Tjaden, Sufian S. Ahmad, Matthias M. Gaida, Andrea S. Bauer, Jens Werner, Nathalia A. Giese

**Affiliations:** 1 European Pancreas Centre, Department of General, Visceral and Transplantation Surgery, University Hospital Heidelberg, Heidelberg, Germany; 2 Institute of Immunology, University Hospital Heidelberg, Heidelberg, Germany; 3 Department of Functional Genomics, German Cancer Research Centre, Heidelberg, Germany; 4 Institute of Pathology, University Hospital Heidelberg, Heidelberg, Germany; The Liverpool Cancer Research UK Centre, United Kingdom

## Abstract

*CSPG4* marks pericytes, undifferentiated precursors and tumor cells. We assessed whether the shed ectodomain of *CSPG4* (s*CSPG4*) might circulate and reflect potential changes in *CSPG4* tissue expression (p*CSPG4*) due to desmoplastic and malignant aberrations occurring in pancreatic tumors. Serum s*CSPG4* was measured using ELISA in test (n = 83) and validation (n = 221) cohorts comprising donors (n = 11+26) and patients with chronic pancreatitis (n = 11+20) or neoplasms: benign (serous cystadenoma SCA, n = 13+20), premalignant (intraductal dysplastic IPMNs, n = 9+55), and malignant (IPMN-associated invasive carcinomas, n = 4+14; ductal adenocarcinomas, n = 35+86). Pancreatic p*CSPG4* expression was evaluated using qRT-PCR (n = 139), western blot analysis and immunohistochemistry. s*CSPG4* was found in circulation, but its level was significantly lower in pancreatic patients than in donors. Selective maintenance was observed in advanced IPMNs and PDACs and showed a nodal association while lacking prognostic relevance. Pancreatic p*CSPG4* expression was preserved or elevated, whereby neoplastic cells lacked p*CSPG4* or tended to overexpress without shedding. Extreme pancreatic overexpression, membranous exposure and tissue^high^/sera^low^-discordance highlighted stroma-poor benign cystic neoplasm. SCA is known to display hypoxic markers and coincide with von-Hippel-Lindau and Peutz-Jeghers syndromes, in which p*VHL* and *LBK1* mutations affect hypoxic signaling pathways. *In vitro* testing confined p*CSPG4* overexpression to normal mesenchymal but not epithelial cells, and a third of tested carcinoma cell lines; however, only the latter showed p*CSPG4*-responsiveness to chronic hypoxia. siRNA-based knockdowns failed to reduce the malignant potential of either normoxic or hypoxic cells. Thus, overexpression of the newly established conditional hypoxic indicator, *CSPG4*, is apparently non-pathogenic in pancreatic malignancies but might mark distinct epithelial lineage and contribute to cell polarity disorders. Surficial retention on tumor cells renders *CSPG4* an attractive therapeutic target. Systemic ‘drop and restoration’ alterations accompanying IPMN and PDAC progression indicate that the interference of pancreatic diseases with local and remote shedding/release of *sCSPG4* into circulation deserves broad diagnostic exploration.

## Introduction

Pancreatic cancer is one of the most aggressive therapy-resistant gastrointestinal malignancies. Because of the often late diagnosis, surgery represents a curative option for only 20% of patients, but this does not preclude recidivism or metastasis [1]. Novel diagnostic and therapeutic approaches are urgently needed. Pancreatic cancer is associated with a prominent desmoplastic reaction of prognostic and diagnostic relevance [2]–[4]. In a mouse model of PDAC, depletion of a rare population of the FAP^+^-fibroblasts/pericytes has been found to be sufficient to induce IFNg/TNF-mediated hypoxic (ischemic) necrosis of cancer cells and stroma via vascular damage and alleviated local immunosuppression [5]. Pericytes are mesenchymal cells seeding in the walls of newly formed blood vessels, playing a major role in angiogenesis, and representing one of the possible sources of the stroma-producing myofibroblasts in pancreatic cancer [6]. They are marked by chondroitin sulfate proteoglycan 4 (*CSPG4*/*NG2*), also known to decorate immature precursors of epidermal, neural, and mesenchymal origin [7]–[10].

Transmembrane *CSPG4* functions as a high-affinity receptor for *bFGF*, *PDGF-AA*, alpha3beta1-integrin, galectin-3, and, most prominently, type VI collagen (*COL6*) [11]. The ligand-receptor interaction activates small GTPases; it can provide links to a number of cytoplasmic signaling pathways, enabling dynamic rearrangement of the actin cytoskeleton, altering cell-stratum interactions and promoting the process of migration. *CSPG4* has also been found in certain tumor cells, and has been shown to promote their malignant behavior and to impact the progression of melanoma, glioblastoma, chondrosarcoma and leukemia [11]–[15]. Therapeutic targeting of *CSPG4* in melanoma and glioblastoma appears to yield anti-tumor effects [16]–[18].

The extracellular portion of *CSPG4* (ectodomain) may undergo different post-translational modifications [19], or be shed, assuming that autocrine proteolysis is not blocked by simultaneously produced *COL6*, which contains a Kunitz-type proteinase inhibitor sequence in the a3-chain [20]. The presence of ectodomain in circulation (s*CSPG4*), and its clinical relevance have not yet been evaluated. Although pro-stromal and pro-malignant, *CSPG4* was not yet considered as a pathogenic factor or biomarker in pancreatic cancer; a stroma-rich aggressive malignancy [3], [21]. By studying the patients with non-neoplastic, benign and malignant pancreatic disorders, we sought to determine whether different degrees of malignant or desmoplastic transformation modify patterns of *CSPG4* expression in tissues or circulation, and whether it might bear diagnostic or pathogenic relevance.

## Materials and Methods

### Serum and Tissue Sampling

The analyses included the pancreatic biopsies and sera from donors and patients with chronic pancreatitis or different variants of exocrine pancreatic tumors: benign, premalignant and malignant. The study was approved by the Ethics Committee of the Faculty of Medicine, University of Heidelberg, Germany (Vote 301/2001 and 159/2002) and performed with patients’ written informed consent and in compliance with institutional regulations. Freshly removed tissues were flash-frozen in liquid nitrogen for RNA and western blot profiling, or fixed in paraformaldehyde solution for 12–24 h prior to paraffin embedding for histological analysis. Serum s*CSPG4* was measured using ELISA in test (n = 83) and validation (n = 221) cohorts comprising donors (n = 11+26) and patients with chronic pancreatitis (CP, n = 11+20) or tumors: i) benign (serous cystadenoma SCA, n = 13+20), ii) premalignant (intraductal papillary mucinous neoplasms with low/intermediate-grade dysplasia (IPMN^dys^, n = 8+36) and with high-grade dysplasia/carcinoma *in situ* (IPMN^tis^, n = 1+19)), and iii) malignant (IPMNs with an associated invasive carcinoma (IPMN^inv^, n = 4+14) and ductal adenocarcinomas including anaplastic (n = 4+7), adenosquamous (n = 4+11) and PDAC (n = 27+68)). Pancreatic p*CSPG4* expression was evaluated using qRT-PCR (n = 139), western blot analysis and immunohistochemistry. The patients’ characteristics are given in Tables 1 and 2.

**Table 1:** Characterization of the patients and data summary.

					Test cohort		Validation cohort	
					Pancreas p*CSPG4* [mRNA/10 kCPB] n = 139	Sera s*CSPG4*[ng/ml] n = 83	Sera s*CSPG4*[ng/ml] n = 221	
Diagnosis		Condition		Age, yearsmedian [range]	No. patients (female/male) mean±SEM median [IQR]			
**Donor**		Norm		Test: 44 [16–71]Valid: 43 [19–74]	n = 14 (5/9)12±113 [9]–[16]	n = 116.8±1.74.8 [2.5–11.4]	n = 26 (15/11)7.3±0.66.6 [5.2–8.4]	
**CP**, chronic pancreatitis		Inflam-mation		Test: 44 [16–76]Valid: 61 [24–78]	n = 15 (3/12)25±326 [14]–[33]	n = 116.8±3.92.8 [1.4–6.1]	n = 20 (7/13)5.0±0.93.7 [2.6–6.5]	
**SCA**, serous cystadenoma		Benign neoplasm		Test: 61 [38–79]Valid: 60 [44–79]	n = 13 (10/3)159±42113 [37–244]	n = 132.6±0.52.0 [1.9–2.8]	n = 20 (13/7)6.8±1.04.0 [2.3–6.7]	
**Intraductal papillary mucinous neoplasms (IPMN)**								
**IPMN^dys^**, Low- and intermediate-grade dysplasia		Pre-malignant		Test: 60 [41–73]Valid: 65 [42–83]	n = 12 (3/9)20±519 [4]–[29]	n = 84.1±0.94.6 [1.5–5.5]	n = 36 (18/18)4.6±0.34.6 [3.1–5.9]	
**IPMN^tis^,** High-grade dysplasia/carcinoma in situ		Pre-malignant		Test: 74Valid: 65 [41–79]	n.d.	n = 1(−/1)1.8–	n = 19 (8/11)3.7±0.52.8 [2.1–4.8]	
**IPMN^inv^,** with an associated invasivecarcinoma		Malignant		Test: 66 [46–76]Valid: 64 [46–73]	n = 13 (1/12)29±429 [21]–[39]	n = 41.3±0.31.3 [0.7–1.9]	n = 14 (3/11)7.6±1.27.9 [3.9–9.7]	
**Ductal adenocarcinomas (DACs)**								
**AdSq,** adenosquamous carcinoma	Malignant		Test: 65 [51–76]Valid: 69 [41–76]		n = 17 (8/9)39±539 [12]–[49]	n = 46.1±3.13.8 [1.9–12.5]	n = 11 (5/6)3.8±0.53.5 [2.6–5.3]	
**Anaplasti**c, undifferentiatedcarcinoma (G4)	Malignant		Test: 56 [45–74]Valid: 66 [53–75]		n = 10 (4/6)54±2731 [26]–[45]	n = 45.0±2.04.3 [1.7–9.1]	n = 7 (3/4)6.0±1.44.6 [2.7–11.1]	
**PDAC** (G1–G3), pancreatic ductaladenocarcinoma	Malignant		Test: 66 [38–80]Valid: 65 [39–81]		n = 45 (20/25)36±1116 [8]–[39]	n = 272.6±0.41.5 [1.1–4.1]	n = 68 (30/38)5.2±0.73.5 [1.9–6.3]	

**Table 2:** Circulating s*CSPG4* in pancreatic diseases: ROC curve analyses.

Group	Test cohort					Validation cohort				
	n = 83	median	AUC	95% CI	P-value	n = 221	median	AUC	95% CI	P-value
**Donor**	**11**	4.8				**26**	6.6			
** vs**.										
Chronic pancreatitis	**11**	2.8	0.682	0.451–0.912	0.149	**20**	3.7	0.779	0.631–0.927	0.001
SCA	**13**	2.0	0.811	0.629–0.992	0.010	**20**	4.0	0.760	0.606–0.913	0.003
IPMN^dys^	**8**	4.6	0.619	0.359–0.880	0.386	**36**	4.6	0.787	0.677–0.898	<0.0001
IPMN^tis^	[1]	–	–	–	–	**19**	2.8	0.883	0.766–0.990	<0.0001
IPMN^inv^	[4]	–	–	–	–	**14**	7.9	0.515	0.299–0.732	0.876
IPMN^tis+inv^	**5**	1.3	0.927	0.784–1.070	0.008	**33**	4.1	0.727	0.593–0.861	0.003
AdSq DAC	**4**	3.8	0.546	0.198–0.893	0.794	**11**	3.5	0.862	0.735–0.986	<0.001
Anaplastic DAC (G4)	**4**	4.3	0.636	0.304–0.970	0.434	**7**	4.6	0.670	0.386–0.955	0.172
PDAC (G1–G3)	**27**	1.5	0.784	0.623–0.945	0.007	**68**	3.5	0.768	0.675–0.860	<0.0001
- Early PDAC (St. Ia-IIa)	**3**	0.6	0.818	0.501–1.136	0.102	**12**	2.0	0.914	0.763–1.064	<0.0001
- Late PDAC (St. IIb-IV)	**24**	1.5	0.783	0.612–0.953	0.009	**56**	3.9	0.736	0.631–0.841	<0.0001
all IPMNs	**13**	1.9	0.738	0.530–0.946	0.049	**69**	4.5	0.758	0.663–0.854	<0.0001
all DACs**^1^**	**35**	1.9	0.740	0.575–0.906	0.017	**86**	3.6	0.772	0.688–0.856	<0.0001
all malignancies**^2^**	**39**	1.8	0.764	0.605–0.921	0.008	**119**	3.6	0.764	0.683–0.836	<0.0001
all neoplasms**^3^**	**61**	2.0	0.755	0.600–0.911	0.007	**175**	3.8	0.765	0.694–0.836	<0.0001
all pancreatic disorders	**72**	2.0	0.748	0.589–0.899	0.001	**195**	3.8	0.767	0.698–0.835	<0.001
**Chronic pancreatitis**	**11**	2.8				**20**	3.7			
** vs.**										
SCA	**13**	2.0	0.584	0.334–0.833	0.487	**20**	4.0	0.502	0.320–0.685	0.978
IPMN^dys^	**8**	4.6	0.563	0.295–0.830	0.650	**36**	4.6	0.549	0.379–0.717	0.550
IPMN^tis^	[1]	–	*–*	*–*	*–*	**19**	2.8	0.607	0.425–0.789	0.255
IPMN^inv^	[4]	–	*–*	*–*	*–*	**14**	7.9	0.732	0.552–0.911	0.023
IPMN^tis+inv^	**5**	1.3	0.773	0.541–1.004	0.089	**33**	4.1	0.537	0.378–0.695	0.653
PDAC	**27**	1.5	0.620	0.415–0.823	0.253	**68**	3.5	0.563	0.434–0.692	0.392
- Early PDAC (St. Ia-IIa)	**3**	0.6	0.712	0.326–1.098	0.276	**12**	2.0	0.752	0.564–0.939	0.019
- Late PDAC (St. IIb-IV)	**24**	1.5	0.619	0.407–0.830	0.270	**56**	3.9	0.523	0.386–0.659	0.764
all IPMNs	**13**	1.9	0,566	0.328–0.805	0.582	**69**	4.5	0.543	0.397–0.689	0.559
all DACs**^1^**	**35**	1.9	0.567	0.367–0.765	0.511	**86**	3.6	0.545	0.420–0.672	0.534
all malignancies**^2^**	**39**	1.8	0.592	0.395–0.788	0.354	**119**	3.6	0.522	0.399–0.645	0.753
all neoplasms**^3^**	**61**	2.0	0.570	0.374–0.766	0.462	**175**	3.8	0.505	0.380–0.630	0.938
**IPMN^dys^**	**8**	4.6				**36**	4.6			
** vs.**										
IPMN^tis+inv^	**5**	1.3	0.850	0.638–1.062	0.041	**33**	5.4	0.528	0.386–0.670	0.692
IPMN^tis^	**–**	–	–	–	–	**19**	2.8	0.689	0.534–0.843	0.022
IPMN^inv^	**–**	–	–	–	–	**14**	7.9	0.691	0.507–0.874	0.038
**IPMN^tis^**	[1]					**19**	2.8			
** vs.**										
IPMN^inv^	[4]	–	–	–	–	**14**	7.9	0.791	0.631–0.952	0.005

Grouping of the tumors in: **1** (all DACs): AdSq, Anapl, PDAC; **2** (all malignanices): AdSq, Anapl, PDAC, IPMN^inv^; **3** (all neoplasms): SCA, IPMNs, DACs.

### Cell Cultures, Media, Antibodies

Nine DSMZ-certified pancreatic cancer cell lines (Aspc1, Bxpc3, Capan1, Colo357, HS766T, MiaPaca2, Panc1, SU8686, T3M4) and the cervical carcinoma HeLa cell line (positive control for western blot and ELISA analyses) were cultured in RPMI medium supplemented with 10% fetal bovine serum (FBS; PAA Laboratories GmbH, Pasching, Austria). Primary pancreatic stellate cells (PSC) were obtained through the outgrowth method of Bachem et al. [22], cultured in low glucose DMEM/F12 (1:1) medium supplemented with 20% FBS and propagated for up to 8 passages as previously described [23], [24]. Immortalized human pancreatic ductal epithelial cells (HPDE) were received as a gift [25], and cultured in serum-free keratinocyte medium, supplemented with 5 ng/ml recombinant epidermal growth factor (r*EGF*) and 50 µg/ml bovine pituitary extract (Life Technologies GmbH, Darmstadt, Germany).

The panel of primary antibodies included the mouse monoclonal anti-*CSPG4* antibody raised against melanoma cells (LHM2 clone; MAB2585; R&D Systems/RnD, Minneapolis, MN, USA), the rabbit polyclonal anti-*CSPG4* antibody raised against recombinant core protein (H-300; sc-20162; Santa Cruz Biotechnologies/SCBT, Santa Cruz, CA, USA), and the mouse anti-collagen VI *(COL6)* antibody (64CH11 clone, ab49273; Abcam, Cambridge, UK). The secondary antibodies and isotype controls used for immunoblotting, immunohistochemistry, immunofluorescence, and FACS analyses are indicated in the respective sections.

### siRNA Transfection and Functional Studies

To confirm the specificity of the anti-*CSPG4* antibodies and to evaluate the functional relevance of p*CSPG4* in pancreatic cancer, we used siRNA-based knock-downs. Cells were grown up to 50–70% confluence and transfected using the HiPerFect transfection reagent (Qiagen GmbH, Germany) at 10 nM with duplex oligonucleotides: siRNA set 1 (sense: CGG UGA GGG AUG UAA AUG Att; antisense: UCA UUU ACA UCC CUC ACC Gtg), siRNA set 2 (sense: AAA UCU CCG UGG ACC AGU Att; antisense: UAC UGG UCC ACG GAG AUU Ucc) (Ambion Applied Biosystems, USA), or control siRNA set (AAT TCT CCG AAC GTG TCA CGT) (Qiagen, Hilden, Germany) for 48 hours.

To evaluate the effect of *CSPG4* gene silencing on cell functions, proliferation, migration, and invasiveness, the cells were treated with control or *CSPG4*-specific siRNA and analyzed through MTT-based growth assay, scratch test, and Matrigel-based invasion assay, using the standard techniques reported elsewhere [23], [24].

### Induction of Hypoxia

Pancreatic cell lines were grown up to 70% confluence, transferred to the modular incubator chamber (Billups-Rothenberg Inc., Del Mar, CA, USA), flashed for 30 min with the hypoxic gas mixture (0.74% O_2_ and 9.9% CO_2_ in N_2_), and incubated in the closed unit for 3 h or 48 h at 37°C. The same procedure was performed without exposure to hypoxic gas to obtain a normoxic control.

### FACS Analysis

Pancreatic cell lines were suspended in FACS Buffer (2% FBS in PBS), blocked with FcR Blocking Reagent (Miltenyi Biotech GmbH, Begrisch Gladbach, Germany), and incubated for 20 min with mouse anti-*CSPG4* antibody (LHM2 clone; RnD) at room temperature, or IgG1 isotype control (SCBT). We used directly labeled phycoerythrin (PE)-conjugate or unlabeled antibody with subsequently added anti-mouse AlexaFlour488-conjugate. Measurements of expression under normoxic and hypoxic conditions were performed using the FACScan and LSR flow cytometers (Becton Dickinson & Co, Franklin Lakes, NJ, USA).

### Enzyme-Linked Immunosorbent Assay (ELISA)

Determination of the total *CSPG4* protein in biofluids (sera and media conditioned by cell lines) was performed using commercial quantitative sandwich enzyme immunoassays (ELISA kits). The test cohort was evaluated in 2011–2012 using an ELISA kit manufactured by USCN Life Science Inc. (Wuhan, P. R. China; Human Melanoma Associated Chondroitin Sulfate Proteoglycan/MCSP assay; Cat.# E91134Hu). This company became part of the Cloud-Clone Corp. (Houston, TX, USA) and continued to sell the kit under Cat.# SEB134Hu. It should be noted that Cloud-Corp revised the instruction manual for this kit as of August 12, 2013, relabeling the vial with the standard stock as 100 ng/ml, rather than the 10 ng/ml previously given, and introducing a 1:10 dilution step to obtain 10 ng/ml to be used for consecutive serial 1:2 dilutions. We purchased this kit for independent validation studies in October–December 2013 and first performed control retesting of randomly chosen ‘old’ samples. This procedure delivered log-higher s*CSPG4* values, i.e., 5 ng/ml instead of 0.5 ng/ml. Therefore, for presentation, we re-scaled all original measurements of the test cohort by a factor of 10.

As a control, we i) confirmed s*CSPG4*-identity of the standard by performing western blot analysis with H-300/SCBT antibody (not shown), ii) proved the titratability of the sera samples (not shown), and iii) validated the findings with a different ELISA kit (results in the main text). For the latter, we randomly choose samples to represent each tested group and measured s*CSPG4* in 64 sera with a commercial kit manufactured by CUSABIO (CSB-EL006076HU; Wuhan, PRC). The USCN/Cloud-Clone kit employed a recombinant N-terminal fragment of a protein (Leu16-Ser350) as an ELISA standard and immunogen to produce mouse monoclonal (capture) and goat polyclonal (detection) antibodies; CUSABIO - a full-length protein expressed in eukaryotic cells. To enable colorimetric reaction, both USCN/Cloud-Clone and CUSABIO kits used avidin-labeled detection antibodies, streptavidin-HRPO conjugate, and TMB substrate. HeLa-supernatant served as positive control for both ELISAs and delivered values of 11.0 ng/ml and 6.0 pg/ml for Cloud-Clone (standard range: 0–10 ng/ml) and CUSABIO (standard range: 0–1600 pg/ml) immunoassays, respectively. Thus, both ELISAs detected a native human s*CSPG4* ectodomain, albeit most probably its different epitopes.

### Expression Profiling of Tissues

Sentrix Human-6v3 Whole Genome Expression BeadChips (Sentrix Human WG-6; Illumina) were used to identify genes expressed in pancreatic cancer tissue. To synthesize first and second strand cDNA and amplify biotinylated cRNA from the total RNA we used an Illumina TotalPrep RNA Amplification Kit. Hybridization to the BeadChip was performed without modification according to the manufacturer’s instructions. A maximum of 10 µl cRNA was mixed with a 20 µL GEX-HYB hybridization solution. The preheated 30 µl assay sample was dispensed onto the large sample port of each array and incubated for 18 hours at 58°C. Following hybridization, the samples were washed according to the protocol and scanned with a Bead Array Reader (Illumina, San Diego, CA). Raw data were exported from the Bead studio software to R. The data were quantile normalized and log2 transformed. The data have been uploaded to ArrayExpress under accession number E-MTAB-1791.

### Real-time Quantitative Polymerase Chain Reaction

All reagents and equipment for mRNA/cDNA preparation were supplied by Roche Applied Science (RAS, Mannheim, Germany) [23], [24], [26]. mRNA was prepared by automated isolation using a MagNA Pure LC instrument and isolation kit. cDNA was prepared using the First Strand cDNA Synthesis Kit for RT-PCR (AMV) according to the manufacturer’s instructions. Real-time PCR was performed with the LightCycler and *CSPG4* kit supplied by Search-LC (Heidelberg, Germany). The number of *CSPG4* transcripts in the samples was normalized to the expression of the housekeeping gene, cyclophilin B. The final data are presented as the number of transcripts per 10,000 CPB copies (10 kCPB).

### Western Blot Analysis (Immunoblotting)

Cells and tissues were lysed in RIPA-Buffer supplemented with proteinase inhibitors (complete mini, RAS) [23]. 30 µg-protein samples were separated using the NuPAGE Gel system (4-12% Tris-Glycine gel; Invitrogen, Karlsruhe, Germany) and transferred to a nitrocellulose membrane (BioRad, Munich, Germany). Blots were blocked with 5% milk powder in TBS, incubated with mouse LHM2 or rabbit H-300 anti-*CSPG4* antibodies overnight at 4°C, followed by the peroxidise-conjugated secondary anti-mouse or anti-rabbit antibodies for 1 hour, and exposed to a chemiluminescence detection kit (all reagents from ECL Amersham; GE Healthcare Europe GmbH, Freiburg, Germany). For equal loading control analysis, the membrane was exposed to stripping buffer (Thermo Scientific, Rockford, IL, USA) for 30 min at 37°C, and then incubated with an antibody against GAPDH (36 kDa) or beta-actin (42 kDa) (SCBT). Commercial SK-MEL-28 melanoma cell lysate (SCBT) and freshly prepared HeLa cells lysate were used as positive controls for *CSPG4* detection.

### Immunohistochemistry

Three micrometer-thick paraffin-embedded tissue sections were immunostained using a peroxidase-labeled polymer and Envision detection system (Dako GmbH, Hamburg, Germany) to visualize the binding of primary antibodies [3], [23]. Briefly, antigens were retrieved by boiling the tissue sections in 10 mM citrate buffer for 10 min in a microwave oven, then the endogenous peroxidase activity was blocked with 3% hydrogen peroxide in methanol for 10 min, and the nonspecific binding was blocked with Power Block (BioGenex Laboratories, Fremont, CA, USA). Sections were incubated with mouse LHM2 (RnD) or rabbit H-300 (SCBT) anti-*CSPG4* antibodies at 4°C overnight. Normal mouse IgG1s or rabbit IgGs (Dako) were used as negative controls. The HRP-labeled anti-mouse or anti-rabbit polymers (Dako) were applied at room temperature for 45 min. The reaction product was visualized using a DAB chromogen/H_2_O_2_ substrate mixture, resulting in brown staining, and sections were counterstained with Mayer’s haematoxylin. Slides were analyzed using an Axioplan-2 imaging microscope (Carl Zeiss Microscopy GmbH, Jena, Germany).

### Immunofluorescence and Confocal Laser Microscopy

Paraformaldehyde-fixed tissues and cell lines additionally treated with 0.3% Triton X-100 for 5 min were incubated with blocking solution (2% FBS, 2% bovine serum albumin, 0.2% gelatine in PBS), and exposed to mouse LHM2 anti-*CSPG4* antibody (single staining) or to a combination of the rabbit anti-*CSPG4* (H-300) and mouse anti-*COL6* (64CH11) antibodies (double staining). After overnight incubation at 4°C, the secondary antibodies were applied at room temperature for 1 h: anti-mouse AlexaFluor488 (green)-conjugated IgG (single staining) or anti-rabbit Cy2 (green)-conjugate and anti-mouse Cy3 (red)-conjugate (double staining). Normal mouse IgG1 and IgG2a or rabbit IgGs were used as negative controls. The nuclei were visualized through DAPI. Slides were analyzed using an Axioplan-2 imaging microscope. Double stained slides were also viewed with a confocal laser scanning microscope (TCS-SP, Leica Microsystems GmbH, Wetzlar, Germany; courtesy of Dr. N. Brady; Bioquant/DKFZ).

### Statistical Analysis

**Figure 1:**
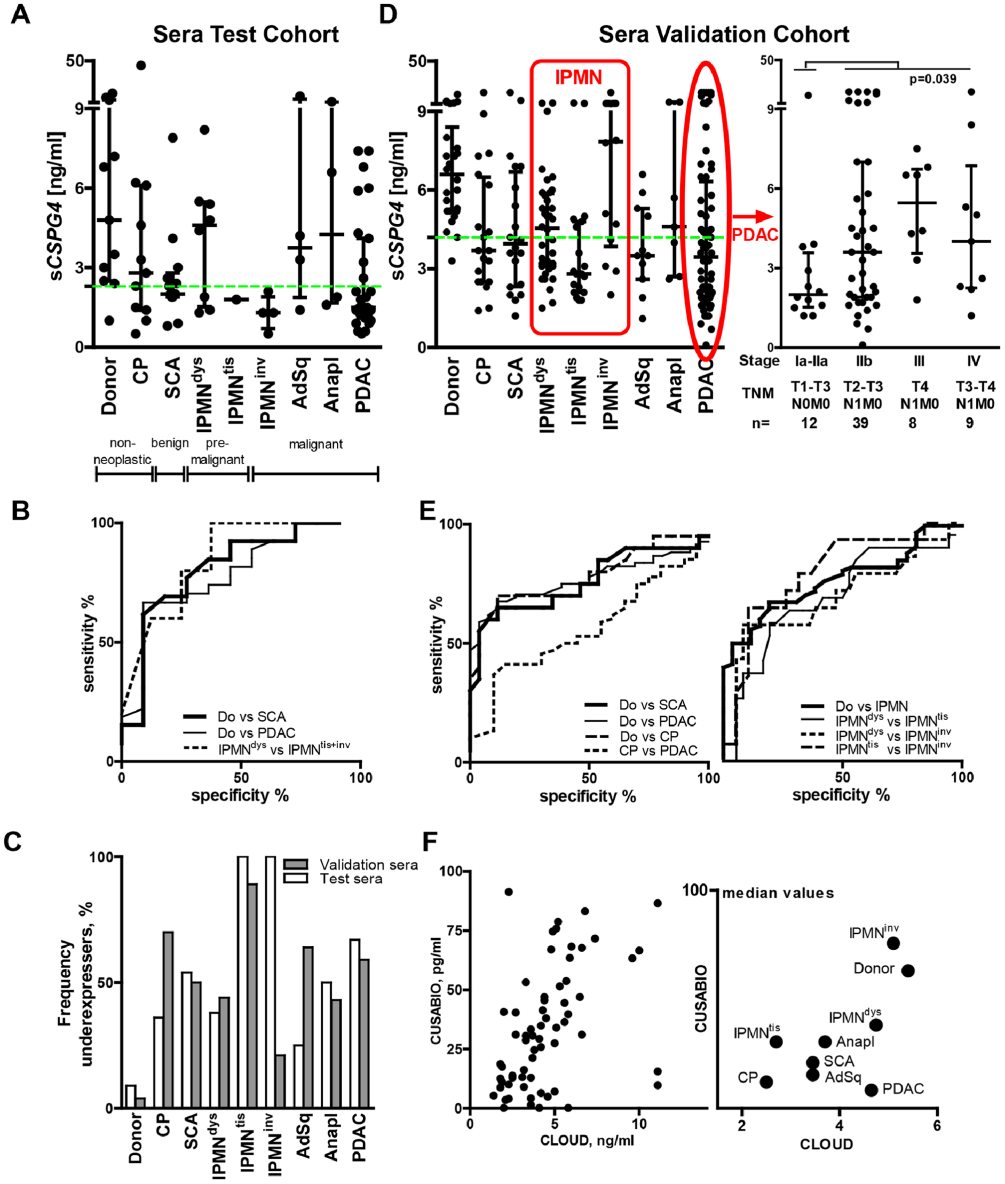
Reduction of serum *CSPG4* (s*CSPG4*) in pancreatic diseases and its selective preservation following the ‘drop and restoration’ pattern in advanced IPMN and PDAC. (**A**) Systemic levels of s*CSPG4* were determined by ELISA in sera (USCN/Cloud-Clone Corp. kit; n = 83) of healthy volunteers and patients with chronic pancreatitis (CP), serous cystadenoma (SCA), premalignant (dysplastic IPMN^dys^ and IPMN^tis^), and malignant (IPMN with an associated invasive carcinoma, IPMN^inv^) forms of intraductal papillary-mucinous neoplasm, as well as in ductal (PDAC), adenosquamous (AdSq) and anaplastic (Anapl) carcinomas. The data are summarized to show individual values, median level, and interquartile range (IQR). (**B**) The ROC analyses of the ELISA data showed the discriminatory power of s*CSPG4* levels for patients with different pancreatic diseases. The dotted green line represents the median level of the particular cohort used to estimate the frequency of the *sCSPG4* underexpressers in the panel (**C**), as detailed in Materials and Methods. (**D–E**) Validation of ELISA findings in an independent cohort (n = 221). Red-bordered areas indicate subgroups depicting IPMN and PDAC progression. The patients’ characteristics and results of statistical analyses are presented in the main text and Tables 1 and 2. (F) Validation of ELISA findings with a different ELISA kit (CUSABIO; n = 64).

**Figure 2:**
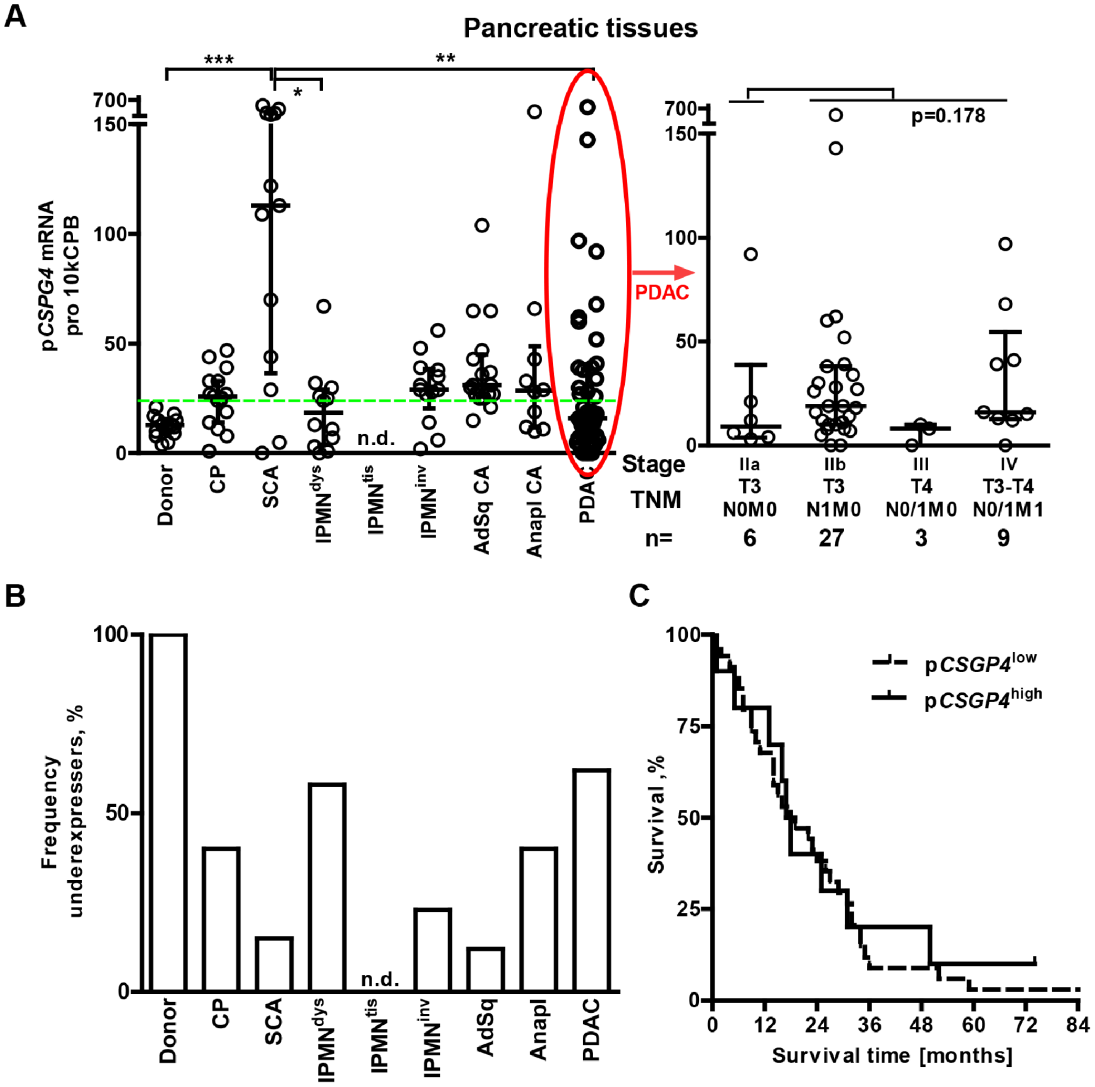
Maintenance of p*CSPG4* mRNA expression in pancreatic tissues. (**A**) The p*CSPG4* copy number was measured using qRT-PCR in pancreatic tissues (n = 139) and normalized to that of the housekeeping gene cyclophilin B (CPB). It is presented as the amount of transcripts per 10 kCPB copies. An elevated p*CSPG4* mRNA level was observed in all groups compared to donors (p-values <0.01; Mann-Whitney tests), except IPMN^dys^ (p = 0.47) and PDAC (n = 0.20). Kruskal-Wallis and Dunn’s test analysis, comparing multiple groups within the entire cohort, established an overexpression of p*CSPG4* in serous cystadenoma, SCA (*p<0.05, **p<0.01 and ***p<0.001 significance level). The dotted green line represents the median level used to estimate the frequency of the underexpressers in the panel (**B**). (**C**) Kaplan-Meier survival curves for p*CSPG4* ^low^ and p*CSPG4* ^high^ (upper quartile; over 38 copies/10 kCPB) groups.

Statistical analyses and graphical data presentation were performed using GraphPad Prism 5 (Graph Pad Software, La Jolla, CA). The quantitative variables are presented as dots showing raw values and bars showing the median and interquartile range (IQR). The significance of differences between groups was assessed using non-parametric methods (Mann-Whitney test for two groups and Kruskal-Wallis with Dunn’s test for multiple groups). *CSPG4*-underexpression was determined as a negative value obtained upon log2-transformation of the individual measurements first normalized to the total median level. The respective median levels are depicted as green lines in Figs. 1A, 1D and 2A, and show 2.3 ng/ml for the s*CSPG4* test cohort (n = 83), 4.2 ng/ml for the s*CSPG4* validation cohort (n = 221), and 24 copies/10 kCPB for the p*CSPG4* mRNA cohort (n = 139). The discriminatory potential of the serum s*CSPG4* was determined by means of receiver operating characteristic (ROC) curves. The regression analyses were performed using the IBM SPSS19 statistics software (IBM Co, Armonk, NY, USA). The overall survival rate from the date of initial surgery was estimated using the Kaplan–Meier method and differences between survival curves were analyzed using the log-rank test. Two-sided P values were computed and a difference was considered statistically significant at p≤0.05 (*), p<0.01(**) or p<0.001(***).

## Results

### Preferential Reduction of Serum *CSPG4* (s*CSPG4*) in Pancreatic Diseases

We suggested that extracellular shedding of *CSPG4* might normally occur in human tissues and lead to the release of the soluble ectodomain (s*CSPG4)* into circulation. Thus, propagation of *CSPG4-*expressing pericytes and tumor cells might suffice to raise systemic levels of s*CSPG4* in patients with malignancies. Using an ELISA kit (USCN/Cloud-Clone Corp.), we tested sera of healthy volunteers (n = 11) and patients with the four most common pancreatic diseases (n = 72): chronic pancreatitis (CP), cystadenoma (SCA), intraductal papillary mucinous neoplasms (IPMN^dys^, IPMN^tis^, and IPMN^inv^ with low/intermediate-, high-grade dysplasia, and with invasive carcinomas), and ductal malignancies (PDAC, adenosquamous and anaplastic) (Table 1). This first-ever evaluation of circulating s*CSPG4* established that the normal median concentration was 4.8 ng/ml, and the mean 6.8 ng/ml. Unexpectedly, s*CSPG4* levels were lower in sera of patients with pancreatic disorders (Fig. 1A; p = 0.01). The drop in s*CSPG4* had already been seen in the inflammatory CP group (n = 11), but that altered level did not show a statistically significant difference from either normal (p = 0.158) or neoplastic ones (p-values ranging from 0.100 to 0.690). s*CSPG4* reduction reached significance in the neoplastic group (n = 61, p = 0.008), mostly due to a remarkable decline in patients with benign SCA (n = 13, p = 0.011), IPMN^tis+inv^ carcinomas (n = 5, p = 0.009), and PDAC (n = 27, n = 0.007). The s*CSPG4* differences observed discriminated these patients from donors–but not CP patients–by means of ROC curve analysis (Fig. 1B; Table 2, test cohort). Notably, patients’ s*CSPG4* values were not ubiquitously down-regulated; some patients appeared to maintain normal s*CSPG4* levels while an increased number presented extremely low values, raising the frequency of underexpressers (Fig. 1C).

Remarkably, IPMN carcinomas (IPMN^tis+inv^; one case of carcinoma *in* s*itu/*Tis and four cases of invasive tumors) were associated with the strongest drop in s*CSPG4* (p = 0.009), whereas premalignant IPMN^dys^ cases showed the best level of s*CSPG4* retention (n = 8, p = 0.471). The s*CSPG4*-distinction among IPMNs (IPMN^dys^ vs. IPMN^tis+inv^, p = 0.047) appeared to suffice for ROC discrimination of these entities (Fig. 1B; Table 2, test cohort). This indication, however, should be viewed with caution due to the inability of such a small cohort to represent heterogeneity of IPMNs [27]–[30].

Among ductal malignancies (n = 35, p = 0.018), the s*CSPG4* drop was more prominent in patients with differentiated adenocarcinoma (PDAC G1-G3: n = 27, n = 0.007) than in less differentiated anaplastic (G4: n = 4, p = 0.473) and adenosquamous (n = 4, p = 0.845) variants. Nevertheless, the degree of tumor cell differentiation did not determine s*CSPG4* variance (p = 0.125 and p = 0.148 compared to PDAC, respectively; p = 0.40 for PDAC G2 vs. G3). In contrast, s*CSPG4* in sera of patients with metastatic PDAC (UICC Stage IV: n = 5 N1M1, p = 0.610) was significantly higher than in those with non-metastatic PDAC (UICC Stages I–IIb: n = 22, with 3 N0M0 and 19 N1M0, p = 0.003), without exceeding normal amounts. s*CSPG4* level in PDAC, however, did not have prognostic relevance (survival time analyses using Cox regression [p = 0.509] and the Kaplan-Meier method comparing s*CSPG4* ^low^ and s*CSPG4* ^high>Q3^ groups with median survival times of 20 and 22 months, log-rank test p = 0.450).

Thus, the test cohort data supported our hypothesis that s*CSPG4* would be found in circulation. It also indicated that the development of pancreatic diseases may reduce systemic s*CSPG4* levels. Seemingly normal levels in certain subgroups of the patients could be attributable either to patient-specific preservation or to cell/stage-specific restoration. The latter variant means that in pancreatic disease, s*CSPG4* changes might generally follow a ‘drop and selective restoration’ pattern.

### Independent Validation of the ‘Drop and Restoration’ Pattern

Our test cohort represented a selection of 72 patients affected by the four most common pancreatic diseases. As a small sample size can lead to overstatements, we analyzed s*CSPG4* in an independent cohort (n = 221) consisting of 26 donors and 195 patients (Figs. 1D–F, Tables 1 and 2, validation cohort). The normal median level was 6.6 ng/ml and the mean 7.3 ng/ml (no significant difference from the test cohort donors according to the Mann-Whitney test, p = 0.240). Independent validation established the reduction of circulating s*CSPG4* as a statistically significant feature of all pancreatic diseases, including CP (3.7 ng/ml, n = 26, p<0.001).

We took particular care to prove the ‘drop and restoration’ assumption by increasing the number of patients representing different stages of PDAC and IPMN progression. Raising the IPMN cases from 13 (8^dys^, 1^tis^, and 4^inv^) in the test cohort to 69 (36^dys^, 19^tis^, and 14^inv^) in the validation cohort substantiated the s*CSPG4*-distinctivity of IPMNs and highlighted the heterogeneity of IPMN^tis+inv^ carcinomas. Taken as separate entities, non-invasive IPMN^tis^ showed the lowest observed level of s*CSPG4* (2.8 ng/ml), whereas invasive IPMN^inv^ displayed the highest (7.9 ng/ml). Although the validation study relocated non-invasive IPMN^dys^ with low−/intermediate-grade dysplasia from the ‘preserved‘ to the ‘reduced’ position (4.6 ng/ml, n = 36, p = 0.0001), the level was still higher than in IPMN^tis^ with high-grade dysplasia (p = 0.023), but lower than in IPMN^inv^ with associated invasive carcinoma (p = 0.039). These differences confirmed the ROC curve-based discrimination of IPMN entities from the donors as well as from each other (Fig. 1E and Table 2, validation cohort). However, the previously observed distinction between IPMN^dys^ (high) and IPMN^tis+inv^ (low) should preferentially be attributed to the non-invasive IPMN^tis^ group. Therefore, the discrepancy between the IPMN^inv^ profile in the test and validation cohorts (Figs. 1A, C and D) demonstrated most strongly how sample size might impact the conclusion, particularly when studying a disease the morphological classification of which alone does not fully encompass its complexity or heterogeneity, and possibly distinct carcinogenic pathways [27]–[31]. Nevertheless, taken as an adenoma–carcinoma sequence, IPMN’s dys→tis→inv progression appeared to deliver a ‘drop and restoration’ s*CSPG4* pattern, with progressive reduction in the circulation of s*CSPG4* in premalignant stages and compensatory gain in the advanced stage.

In PDAC (n = 68), the reduced s*CSPG4* levels were not distinguishable from those in CP (p = 0.394). However, the PDAC subgroup with early disease (lymph node and/or metastasis-free) showed the lowest median s*CSPG4* value (2 ng/ml; IUCC stages Ia-IIa: T1-3N0M0, n = 12; Fig. 1D, right panel). The value for this subgroup was significantly lower than that for the CP group (n = 0.019), and also the late/advanced PDAC group (3.9 ng/ml; stages IIb–IV: T2-4N1M0-1, n = 56, p = 0.0386). Here, the impact of the nodal but not metastatic status reached statistical significance (for 59 M0 vs. 9 M1 patients, p = 0.520). As in the test cohort, the degree of tumor cell differentiation did not determine s*CSPG4* variance (PDAC G2 vs. G3, p = 0.23; PDAC, anaplastic and adenosquamous variants, p = 0.372).

Overall, the validation study reinforced the positioning of any pancreatic disease as an s*CSPG4*-reducing factor, with certain (aggressive/advanced) malignancies possessing s*CSPG4*-restoring features.

### Validation of the Findings with a Different ELISA Kit

The observed reduction in the s*CSPG4* level could be absolute or relative, i.e., reflecting either the disappearance of proteoglycans from circulation entirely or of recognizable epitopes from the still circulating s*CSPG4*. To address this issue, we re-measured the validation sera with a different ELISA kit. Instead of the N-terminal (Leu16-Ser350) fragment used by USCN/Cloud-Clone Corp., this CUSABIO-manufactured kit employed full-length immunogen to generate monoclonal capture and polyclonal detection antibodies. In the control experiment, both ELISAs measured s*CSPG4* shed in the supernatants by HeLa cells, albeit at different levels: 11 ng/ml by Cloud-Clone and 6 pg/ml by CUSABIO. The sera data also showed ng and pg levels of measurements, with significant correlation of individual s*CSPG4* values in 64 randomly chosen samples (Fig. 1F, left panel; Spearman Rho = 0.52; p<0.0001). Moreover, the plotting of median values confirmed the s*CSPG4*-reducing character of pancreatic diseases (Fig. 1F, right panel). These data suggest that the disappearance of s*CSPG4* from circulation is more likely to occur than a loss of specific epitopes.

Such a drop in circulating s*CSPG4* could be the result of excess degradation or reduced production, occurring more frequently in pancreatic patients than in healthy individuals. A few samples showing discrepant CUSABIO^high^/Cloud^low^ or CUSABIO^low^/Cloud^high^ patterns contradicted the common degradation of circulating s*CSPG4* in pancreatic diseases. Furthermore, in the case of proteolysis, at least one of the cleaved s*CSPG4*-fragments should be recognized by an antibody at the non-cleaved-equivalent level (see Discussion). Alternatively, the reduction of systemic s*CSPG4* could reflect diminished production and/or release in different organs, including a disorganized pancreas.

### Differential Expression of p*CSPG4* in Human Pancreatic Tissues (p*CSPG4*)

To clarify whether observed drops in circulating s*CSPG4* levels were due to reduced intrapancreatic production (p*CSPG4)*, we evaluated p*CSPG4* mRNA expression in normal (organ donor), inflammatory (chronic pancreatitis), and neoplastic pancreata using qRT-PCR (Table 1 and Fig. 2). Surprisingly, the reduction in serum protein levels was contrasted by maintained or elevated pancreatic levels (Fig. 2A). Median p*CSPG4* mRNA values were higher in all patient groups compared to donors, except IPMN^dys^ (n = 12, p = 0.47) and PDAC (n = 45, p = 0.20). The frequency of underexpressers was significantly reduced (Fig. 2B). p*CSPG4* mRNA was overexpressed in 5–25% of malignant specimens and was exceptionally high in almost all benign serous cystadenoma (SCA) biopsies. SCA was the only case of a significant 10-fold increase compared to the donor and CP groups, as well as 2- to 5-fold increase compared to other pancreatic neoplasms (p<0.001); it also had the highest p*CSPG4* ^high^/s*CSPG4* ^low^ discordance. p*CSPG4* mRNA expression in PDAC lesions did not correlate with any of the clinico-pathological parameters, such as age, sex, tumor grading staging, or survival (not shown). In the most frequently diagnosed PDAC subgroup (n = 44, as one patient was lost for follow-up), the Kaplan-Meier analysis showed similar overall survival rates of patients with low and high (upper quartile; >38 copies/10 kCPB) p*CSPG4* expression (Fig. 2C; 18.5 vs. 17.5 months; p = 0.738). Thus, the p*CSPG4* pattern differed among pancreatic diseases, and showed diagnostic but not prognostic relevance.

**Figure 3:**
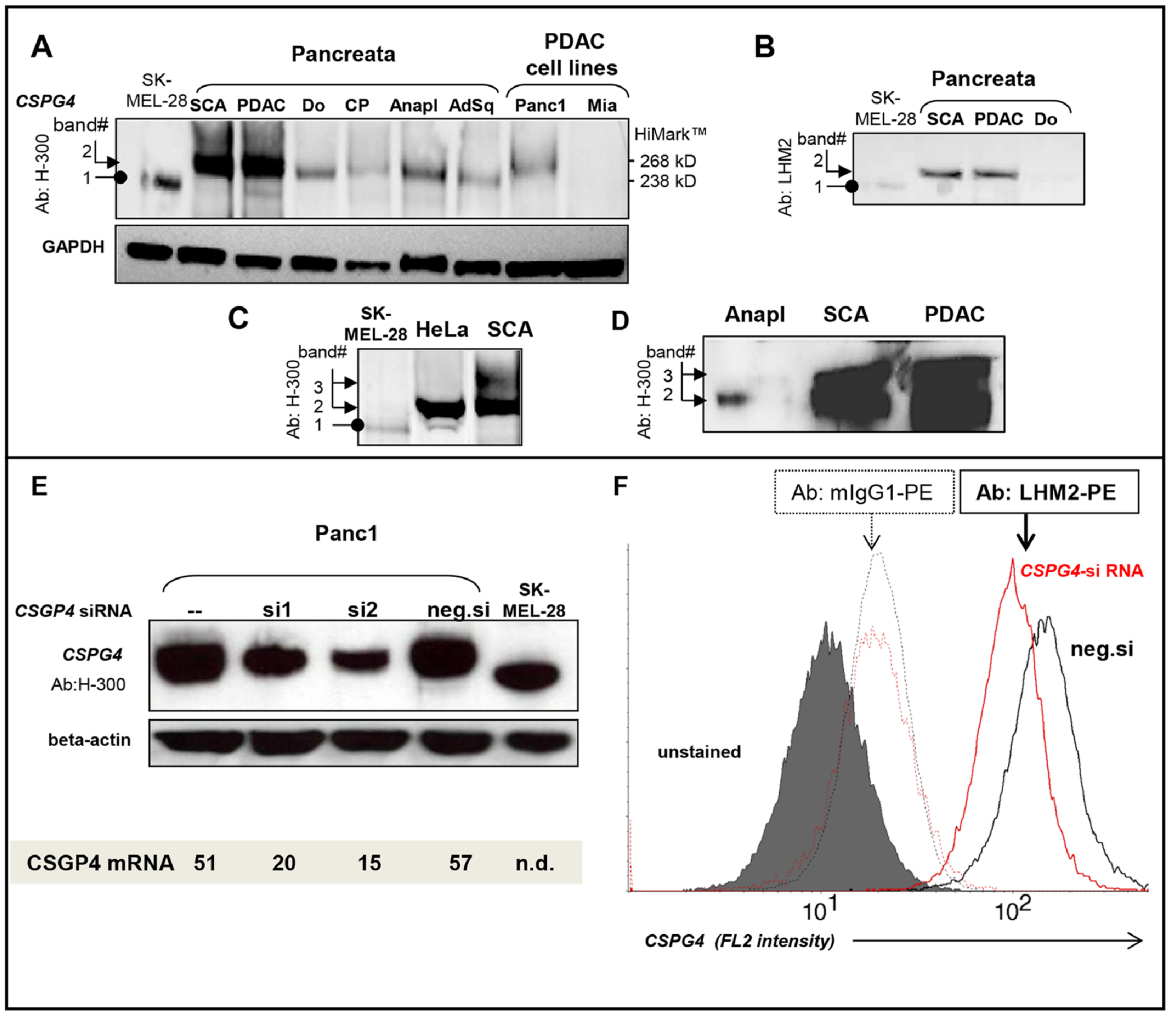
p*CSPG4* protein expression in pancreatic tissues. Immunoblotting confirmed the specificity of polyclonal H-300 (**A**) and monoclonal LHM2 antibodies (**B**), generated against recombinant protein and melanoma-derived antigens, respectively. The images demonstrate differences in size between pancreatic (band-2) and melanoma (SK-MEL-28, band-1) antigens, but not cervical carcinoma (HeLa) antigens (**C**), and reveal the frequent existence of oversized isoforms in SCA and PDAC (band 3, **C–D**). *GAPDH* was used as the loading control. HiMark (Invitrogen) was used as the molecular weight marker. (**E**) Western blot and (**F**) FACS analyses of *CSPG4* siRNA-transfected Panc1 cells further confirmed the specificity of the used antibodies and established the knockdown efficacy of the two siRNA sets (si1 and si2) at approximately 75% after 48 h post transfection, compared to the negative control siRNA (neg.si). QRT-PCR confirmed the efficacy of the knockdowns at the mRNA level.

Although p*CSPG4* mRNA expression was not reduced, it did not exclude the possibility of decreased protein expression. Both core protein-specific polyclonal rabbit H-300 antibodies (immunogen: E.coli-produced recombinant protein; Fig. 3A) and the surface epitope-specific monoclonal mouse LHM2 antibodies (immunogen: melanoma cells A375P and SK23; Fig. 3B) recognized p*CSPG4* protein in pancreatic tissues upon western blot analysis. The molecular weight of normal pancreatic p*CSPG4* protein (band-2, ca. 275 kDa) was higher than that of the melanoma antigen used as a positive control (band-1, ca.250 kDa; lysate of SK-MEL-28 cells). Importantly, inflammatory and neoplastic pancreata retained normal band-2, which was also a major product in ELISA-positive HeLa cells (Fig. 3C). The difference from melanoma was similar to that recently described in human fetal brain and glioblastoma specimens [19]. In the PDAC and SCA samples, this band-2 isoform was overexpressed and frequently accompanied by an additional higher-sized product (band-3, ≥300 kDa, Fig. 3D). Western blot and FACS analyses of siRNA-based *CSPG4* knockdowns in the Panc1 cell line confirmed the *CSPG4* nature of the pancreatic isoform and further validated the p*CSPG4*-specificity of H-300 and LHM2 antibodies (Figs. 3E–F).

### Localization of *CSPG4* in Pancreatic Tissues

**Figure 4:**
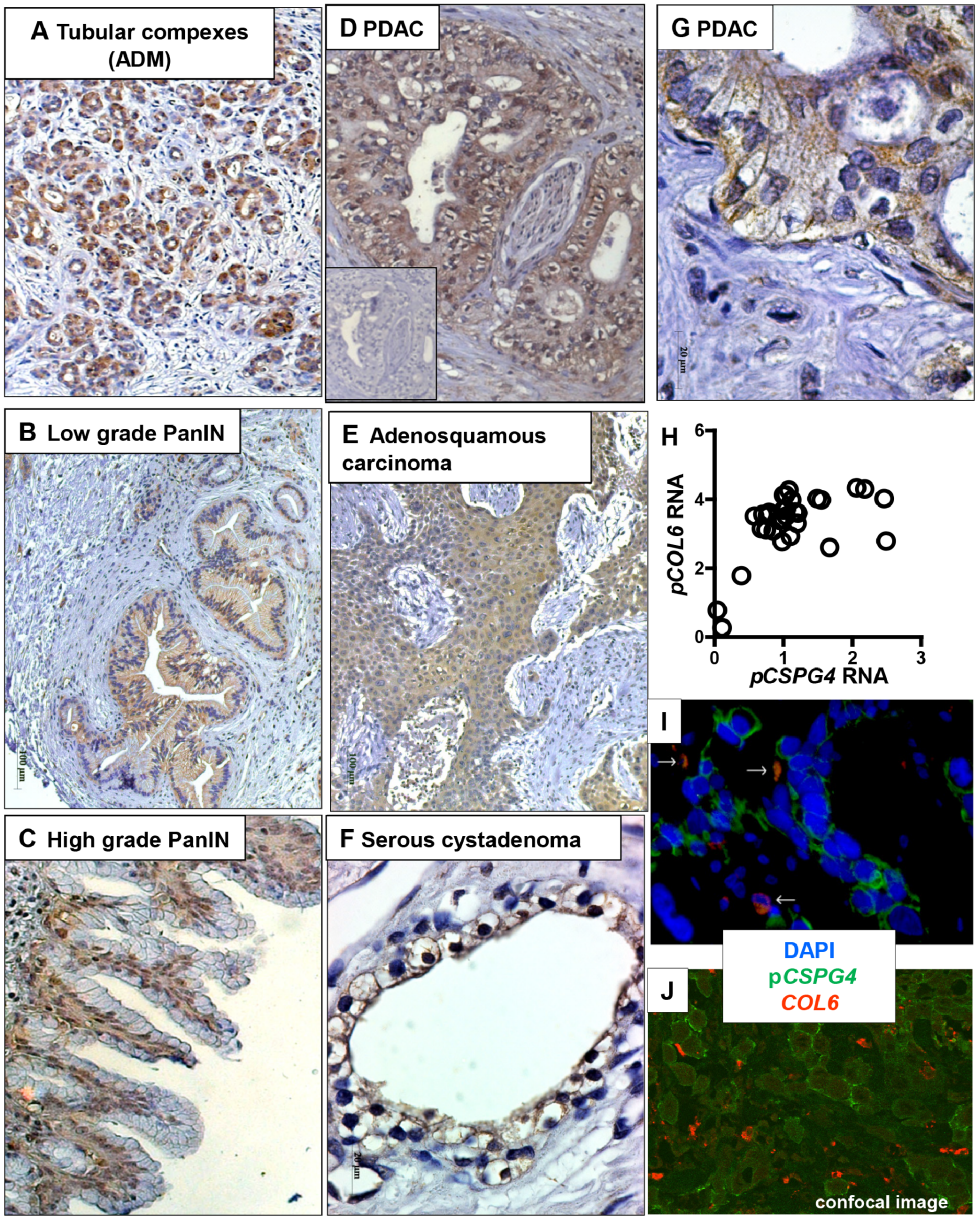
Localization of *CSPG4* in pancreatic tissues. Staining of tissues was performed using mouse LHM2 anti-*CSPG4* antibody, followed by HRP-conjugated anti-mouse polymer and visualization with the Dako Envision system (immunohistochemistry, **A–G**), or using rabbit anti-*CSPG4* antibody (H-300) and mouse anti-*COL6* antibody followed by anti-rabbit-Cy2 and anti-mouse-Cy3 conjugates (immunofluorescence, **I–J**). The antigen-specific antibodies were replaced with isotype IgGs for negative control; the representative image is given as inset in (**D**). Shown (100x–200x) are tubular complexes/acinar-to-ductal metaplasia ADM (**A**), pre-malignant PanIN lesions of low (**B**) and high grade (**C**) in paratumoral areas of PDAC biopsies; perineural invasion of PDAC tumor cells (**D**); squamous compartment in adenosquamous carcinoma (**E**); and high-resolution images (630x) of an epithelium lining of cysts in serous cystadenoma, SCA (**F**) and tumor cells in PDAC lesion (**G**). (**H**) Co-expression of *CSPG4* and *COL6* RNA in pancreatic tissues according to microarray-based measurements. (**I**) Double immunofluorescent staining showed rare co-localization of p*GSPG4* (green) and *COL6* (red; white arrows) in PDAC lesions, and prevalence of *COL6*-free surfaces. The images were routinely recorded using Axiovision Software installed on a Carl Zeiss microscope, and (**J**) confirmed by confocal laser scanning microscopy (TCS-SP, Leica Microsystems, courtesy of Dr. N. Brady, Bioquant, Heidelberg University/DKFZ, Germany).

Despite pancreatic disorganization, RNA and the protein levels of p*CSPG4* were not reduced in diseased organs. To elucidate the reason for maintained/elevated p*CSPG4* expression, we visualized the localization of *CSPG4* in pancreatic tissues. The immunohistochemical evaluation of the various specimens revealed that the cells scattered in islets, stroma and the walls of normal ducts or vessels were the common non-malignant source of *CSPG4* immunopositivity in normal, inflammatory and cancerous pancreata. A proportion of the cells showed co-immunopositivity for chromogranin A (neuroendocrine origin [10]), as did others for desmin, vimentin and *PDGF* receptors (fibroblast-like cells of pericyte origin; not shown). Whereas normal pancreatic ducts lacked *CSPG4*, a strong signal was detected in the tubular complexes emerging among degenerated acini in the paratumoral areas affected by reactive reorganization (Fig. 4A). The same reactive pattern was also found in CP tissues (not shown). Also, premalignant PDAC precursors (PanINs) showed *CSPG4* positivity, from weak/diffuse in low-grade PanINs to strong/basal in higher-grade lesions (Fig. 4B–C).

The malignant lesions lacked islets (and therefore the corresponding *CSPG4* immunoreactivity), but showed irregular focal staining of cancerous ducts in PDAC, strongest in the areas with perineural invasion (Fig. 4D). Diffuse immunopositivity was also observed in squamous elements of adenosquamous carcinoma (Fig. 4E), and in anaplastic carcinomas and invasive IPMN lesions, but not dysplastic IPMN (not shown). Benign SCA showed uniform, prominent accumulation of the *CSPG4* in the epithelial lining of the cysts (Fig. 4F).

The staining of epithelium in benign (p*CSPG4* ^high^/s*CSPG4* ^low^) SCA was exclusively membranous (Fig. 4F); the majority of malignant cells showed diffuse cytoplasmic and/or membranous patterns (Fig. 4G). Type VI collagen (*COL6*) is not only a major interacting partner but also a powerful blocker of *CSPG4* shedding [20]. The microarray-based profiling (Fig. 4H) confirmed the expression of *COL6* in malignant pancreatic lesions [32]. Notwithstanding RNA co-expression, double immunofluorescence und confocal microscopy revealed only occasional co-localization of *COL6* and p*CSPG4,* and the prevalence of *COL6*-free *CSPG4*-positive interfaces (Fig. 4I, white arrows; 4J, confocal microscopy). s*CSPG4* trapping in the extracellular matrix [19] was not observed.

Based on these data, we concluded that the variability of cellular sources allows the pancreas to maintain or elevate p*CSPG4* synthesis; certain types of expanding cells, however, may overexpress *CSPG4* without shedding it.

### Expression and Lack of *CSPG4* Release in Pancreatic Cancer Cells

**Figure 5:**
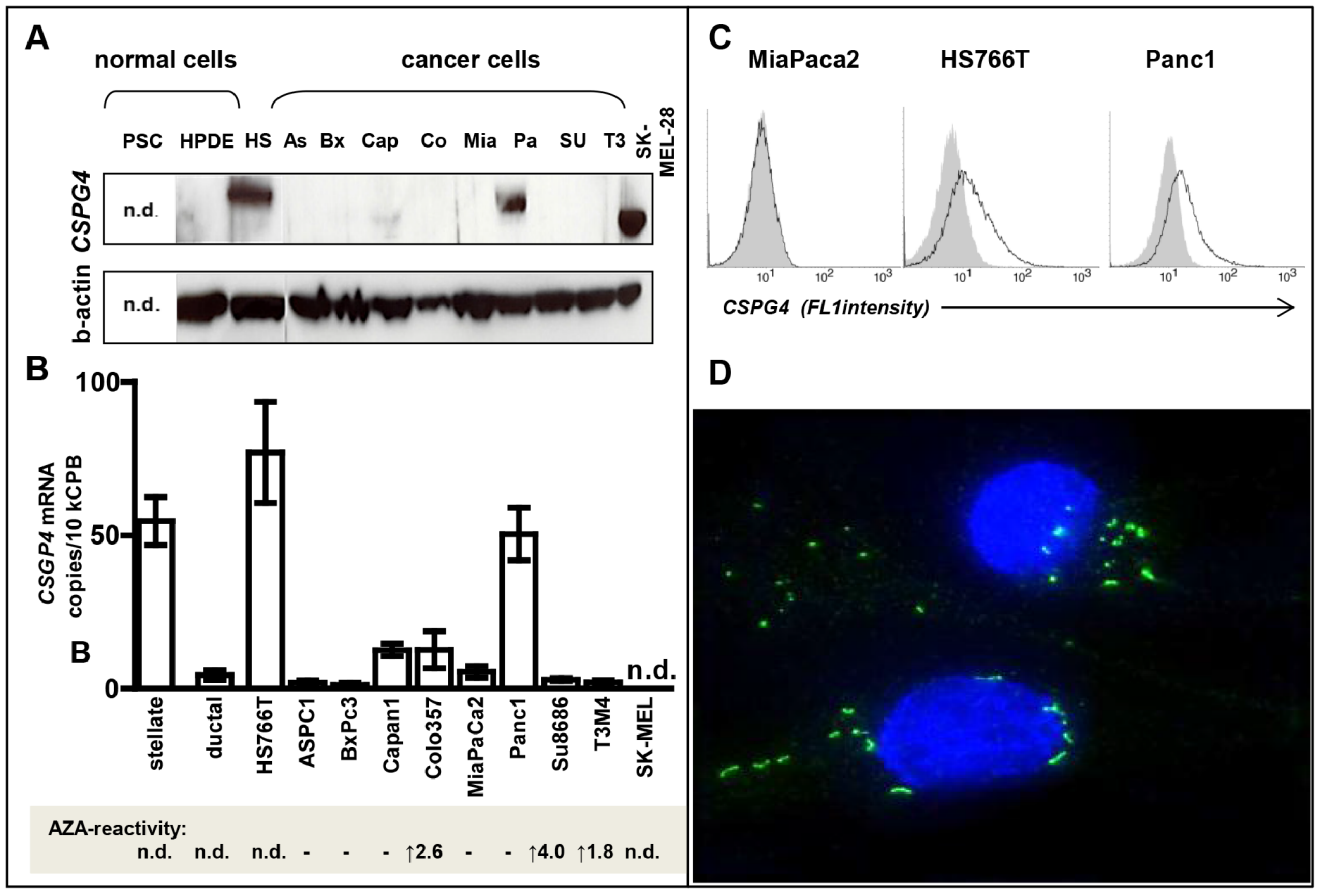
*CSPG4* expression in pancreatic tumor cells. (**A**) Immunoblotting with H-300 antibody detected p*CSPG4* protein in three of nine pancreatic cancer cell lines; shown in relation to melanoma SK-MEL-28 and a normal immortalized ductal epithelial cell line, HPDE. (**B**) QRT-PCR analysis of pancreatic cancer cell lines, normal epithelial HPDE cells and primary myofibroblasts (pancreatic stellate cell, PSC). (**C**) Whereas none of the ELISA kits was capable of detecting s*CSPG4* in supernatants (not shown), FACS analyses with LHM2 and anti-mouse Cy2-conjugate (bold lines) revealed surficial exposure of *CSPG4* by p*CSPG4*-postive Panc1 and HS766T but not p*CSPG4*-negative MiaPaca2. (**D**) The focal accumulation of *CSPG4-*forming dots and short fibrils was visualized by means of immunofluorescence in Panc1 cells. The specificity of staining was confirmed using mouse isotype IgG1 control (not shown).

Among pancreatic cancer cell lines, only 3/9 cultures (Panc1, HS766T and, to a much lesser extent, Capan1) showed an accumulation of p*CSPG4* protein (Fig. 5A), the molecular weight of which, ca. 275 kDa, was higher than in melanoma (band-1) but similar to that in normal and cancerous pancreata, and in the HeLa cell line (band-2, Figs. 3A and 3C). *CSPG4* was not detectable in normal adult ductal cells (HPDE). This pattern was mirrored exactly by the mRNA profile (Fig. 5B), with high constitutive mRNA expression in Panc1 and HS766T comparable to the mRNA level observed in pancreatic stellate cells (PSC) used as a positive control of presumably pericytic origin. In contrast, normal HPDE cells and seven other cancer cell lines showed barely detectable mRNA copies of *CSPG4* (a range of 2–7 copies/10^4^ copies of CPB). The *CSPG4* silencing was only partially alleviated by DNA methylation inhibitor 5-Aza-2′-Deoxycytidine (Aza); the expression was induced in three out of eight cell lines (Colo357, SU8686, T3M4; average increase of 2.7 times; Fig. 5B, lower panel), thus indicating that hypermethylation is only partially responsible for the low level of *CSPG4* transcription in pancreatic tumor cells.

FACS analyses confirmed surficial exposure of *CSPG4* in Panc1 and Hs766T cells; immunofluorescent staining revealed preferentially fibrilar patterns of *CSPG4* distribution (Fig. 5C–D). The secreted/shed form of *CSPG4* was undetectable in the supernatants of different pancreatic cancer cell lines, or in highly expressing Panc1 cells (limit of detection 0.15 ng/ml; data not shown) – in contrast to the strong accumulation of s*CSPG4* in the supernatants of HeLa cells showing Panc1-like band-2 in the p*CSPG4* western blot analysis (Fig. 3C). Apparently, these cells lacked the autocrine shedding mechanism operating in HeLa. Thus, pancreatic cancer cells rarely overexpressed *CSPG4* and tended to retain it on the surface without shedding. Therefore, levels of ectodomain in circulation may not necessarily reflect levels of proteoglycan in tissues, and elevation of p*CSPG4* will not enforce a rise in s*CSPG4*.

### Functional Relevance of p*CSPG4* Expression in Pancreatic Cancer Cells and its Association with Chronic Hypoxia

The lack of prognostic associations, exclusive overexpression of p*CSPG4* in benign neoplasm mainly avoiding malignant transformation (SCA), and relatively rare expression of p*CSPG4* in carcinoma cell lines argued against the attribution of *CSPG4* pro-malignant activity. Thus, the artificial down-regulation of *CSPG4* in p*CSPG4*-expressing cancer cells should not impede their malignant behavior. To prove this, we reduced intrinsic *CSPG4* levels by means of two different siRNA sets in Panc1 and MiaPaca2 cells and compared the effects to those seen in control-siRNA treated cultures. Upon knockdown, MiaPaca2 cells showed complete removal of the few existing RNA copies (although both were undetectable by western blot analysis), and Panc1 showed a 70%-ige reduction in the high *CSPG4* levels at the RNA and protein levels, total and on the surface (Fig. 3E–F). Proliferation, motility and invasion were neither reduced nor promoted (data not shown), questioning any role of *CSPG4* in pancreatic cancer other than as a biomarker.

**Figure 6:**
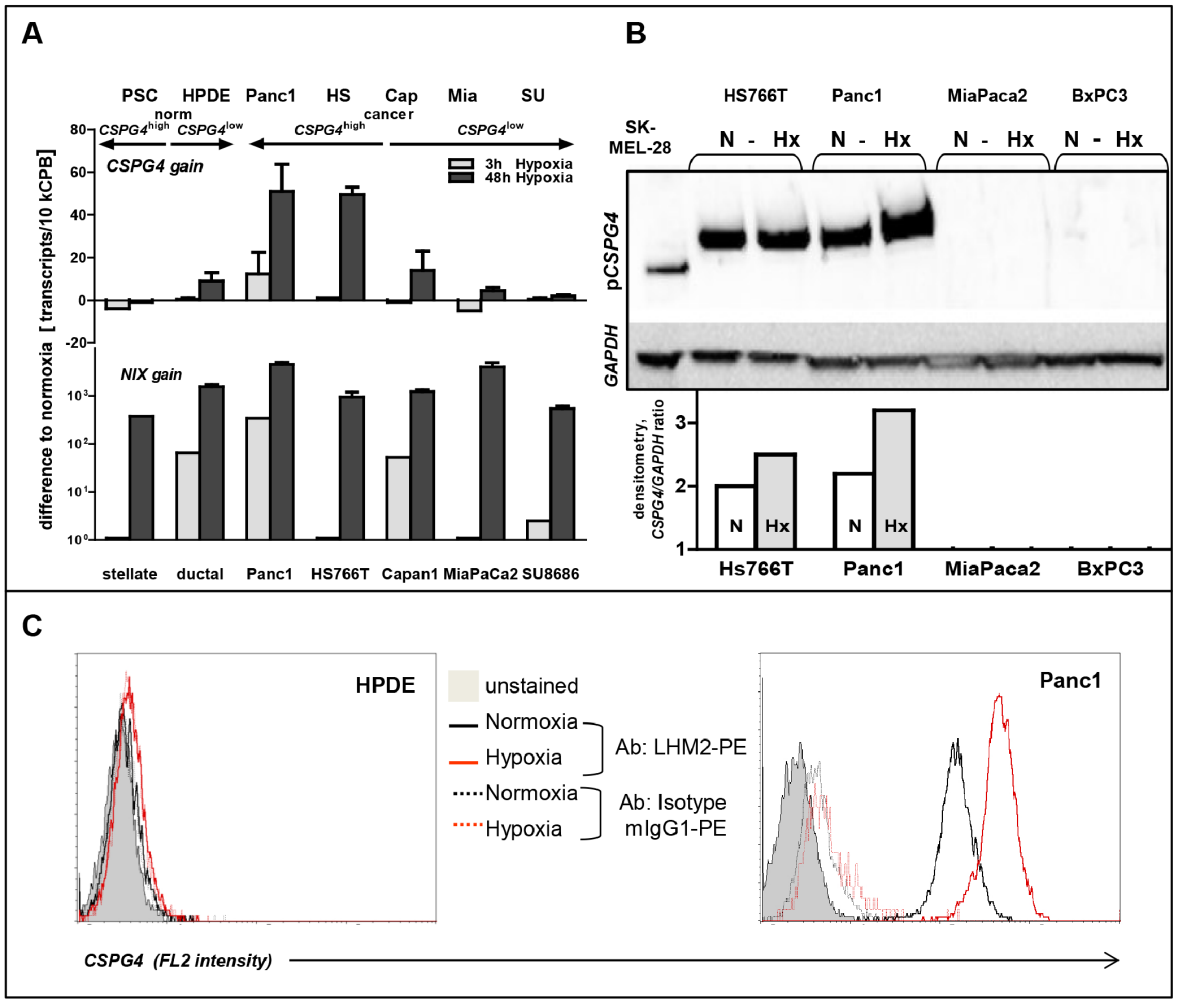
p*CSPG4* as a novel conditional marker of chronic hypoxia in pancreatic tumor cells. Pancreatic normal (stellate and HPDE) and cancer cells grouped according to the level of constitutive p*CSPG4* expression were exposed to hypoxia (0.74% O_2_) for 3 h and 48 h. QRT-PCR (**A**), western blot with densitometric quantification using ImageJ software (**B**) and FACS (**C**) analyses showed up-regulation of p*CSPG4* only in cancer cells with high basal levels of p*CSPG4* at 48 h. In contrast, expression of a known hypoxia-sensitive gene, *NIX*, was strongly up-regulated in all cells.

In addition to benignancy, the most striking feature of SCA is its association with disorders caused by gene mutations that affect hypoxic signaling pathways, such as *pVHL* in von-Hippel-Lindau and *LKB1* in Peutz-Jeghers syndromes [33]–[37]. What is particularly frequent is an association with VHL syndrome, mimicking a hypoxic condition due to the inactivation of the HIFa-suppressing *pVHL* gene. As the SCA cyst epithelium overexpresses a number of hypoxic markers, we sought to determine whether *CSPG4* might represent a novel hypoxia-regulated gene. Normal pancreatic ductal epithelial cells (HPDE) and a panel of cancer cell lines were exposed to <1%-oxygen for 3–18 h (acute hypoxia) or 48–72 h (chronic hypoxia), and profiled using qRT-PCR, western blot and FACS-based methods. Indeed, hypoxic conditioning caused an up-regulation of *CSPG4* expression; however, this was a restricted phenomenon. First, neither *CSPG4* ^low^ HPDE cells nor pancreatic cancer cell lines showed significant elevation of *CSPG4* expression, although hypoxia greatly increased expression of other hypoxic markers, such as *BNIP3*, *EPO* (data not shown), and *NIX* (Figs. 6A–B). Thus, whatever the reason for *CSPG4* silencing in normal ductal cells and the majority of cancer cells, hypoxia was incapable to overcome it. Second, the elevation of *CSPG4* mRNA and protein levels in *CSPG4* ^high^ Panc1 and HS766T cells occurred only upon prolonged (≥48 h) exposure to hypoxia. It should also be noted that hypoxia promoted surface expression of *CSPG4* (Fig. 6C), but it did not induce an accumulation of the shed/secreted form (according to ELISA; not shown). Third, *CSPG4* transcription was not up-regulated in otherwise hypoxia-sensitive *CSPG4* ^high^-primary myofibroblasts/PSCs, the non-transformed cells of non-epithelial origin. This shows that the basal expression alone does not guarantee hypoxic up-regulation. An additional factor is apparently needed.

In combination, these findings indicate that basal expression, mutation/transformation and prolonged oxygen deprivation (physiologic or genetically mimicked) are required for *CSPG4* to show hypoxic inductivity. The pathogenic relevance of this induction remains unclear, because, similarly to normoxic cells, the malignant conduct of hypoxic cells (invasiveness and growth) was not affected by siRNA-based knockdowns (not shown).

## Discussion

In pancreatic cancer, profiling studies have produced a vast number of putative biomarkers and targets, but very few have accomplished clinical translation. This might be related to the common assumption that an over-expressed molecule is a sign of malignant transformation of an adult ductal cell. Aspects such as extra-pancreatic sources, the systemic character of pancreatic disease, and the still unknown origin of pancreatic malignancies are frequently ignored. The impact of pancreatic disorganization (e.g., parenchymal atrophy, desmoplastic reaction) is rarely taken into account and studies often lack other control groups in addition to the healthy donors.

In our view, this study exemplifies the usefulness of employing a panel of different neoplastic entities as a means of critically evaluating the pathogenic and clinical relevance of the particular molecule. The published records predicted the overexpression and pathogenic relevance of *CSPG4* in pancreatic malignancies in line with pro-stromal, pro-angiogenic and pro-malignant activities in other cancers [11]–[15]. Pancreatic expression of p*CSPG4* was maintained or elevated in all tested disorders, but most strongly in stroma-poor benign neoplasm SCA. However, levels of s*CSPG4* ectodomain shed into patients’ circulation dropped. Selective restoration was observed in advanced or aggressive malignancies, whereby – if at all – pancreatic tumor cells demonstrated overexpression without shedding. Apparently, any pancreatic disease is capable of altering *CSPG4* production, whether remote or local, via intrinsic or extrinsic mechanisms. Whereas various cellular sources appear to ensure p*CSPG4* synthesis, release of the ectodomain emerges as the main point of interference (block or promotion).

Although the s*CSPG4* ‘drop and restoration’ pattern is clearly associated with IPMN and PDAC progression, certain limitations in this study preclude immediate discrimination between direct (cell type-dependent) and indirect (disease-dependent; nodal invasion status) contributions. The sample sizes of the test cohort (n = 83) and validation cohort (n = 221) are relatively small in relation to the variety and genetic heterogeneity of pancreatic diseases, particularly IPMNs [27]–[29], [38]. IPMNs, which may have distinct pathways to cancer progression [30], [31], [39], show an intriguing *CSPG4* distinction between non-malignant and malignant entities; only the latter tend to up-regulate both pancreatic and circulating *CSPG4* levels. s*CSPG4*-producing precursors and pericytes are dispersed throughout the body, precluding clear identification of the extra-pancreatic sources to be analyzed. Although we obtained similar results with two different ELISA kits, both assays employed mouse monoclonal antibodies as the capture reagent. The existence of 48 immunologically distinct *CSPG4* isoforms and their diversified expression demonstrated recently [19] indicates that a broad panel of antibodies is required to discriminate between total loss and epitope-specific aberration of s*CSPG4*.

Our observations suggest that pancreatic *CSPG4* is not a pathogenic factor promoting pancreatic carcinogenesis. Rather than a pro-malignant bias, a differentially expressed molecule might be a common attribute of a specific lineage and appear as overexpressed once the population starts to expand. It transpires that pancreatic *CSPG4* overexpression is a robust feature of a stroma-poor benign adenoma SCA. Its known genetic background (VHL syndrome) allows us to suspect the hypoxic inductivity of *CSPG4*. The up-regulation of the *CSPG4* at 48 h but not 3 h of oxygen reduction indicates a link to chronic hypoxia, thus revealing *CSPG4* as a possible target gene of *HIF2a*; that fits the *HIF2a* dependency of VHL syndrome [40], [41]. Furthermore, the increased tumor progression seen in a *KRAS*-driven lung cancer model upon *HIF2a* depletion (i.e., *HIF2a* as a tumor suppressor) is in line with the *CSPG4*’s lack of pro-malignant features [42]. The inability of hypoxia to overcome the *CSPG4* negativity of normal adult ductal cells or 78% of cancer cell lines indicates a lineage-restricted pattern of expression, possibly imposed by hypermethylation, as indicated by our experiments with AZA-treated cell lines.

The pancreas is an extremely heterogeneous tissue and the cellular origins of diverse pancreatic tumors are still unclear [43], [44]. Despite the ductal appearance of most exocrine pancreatic malignancies, the transgenic animal models and extensive histological evaluation of biopsies indicate that it might arise from centroacinar cells, terminal ducts, metaplastic acini, or dormant early progenitors [43]–[49]. Although canonical endocrine, acinar and ductal pancreatic lineages originate from the endoderm, the pancreas is infested with stem cells, mesenchymal progenitors and epithelial populations of ambiguous origin [43], [49], [50]. In particular, centroacinar cells and terminal/intercalated ducts are clearly distinct from other cells in the pancreatic ductal system, and demonstrate dramatic expansion in the setting of chronic epithelial injury [51]. Thus, these cells could form the tubular complexes extending among degenerating acini under physiological stress (e.g., hypoxia [49]), and possibly give rise to SCA under genetic mutation. In addition to pancreatic cysts/SCA, VHL syndrome is associated with the development of renal cysts [52], [53] affecting kidney tubular epithelium known to originate from the mesoderm through mesenchymal-to-epithelial transition (MET) [54], [55]. The preferential association of *CSPG4* with immature (glial) neural, mesenchymal and epidermal progenitors [7]–[10] suggests that *pVHL* mutation might affect either a common early precursor (mesendoderm/primitive streak) or a pancreatic epithelial cell of kidney-like mesodermal morphogenesis. Thus, normal pancreata should shelter scarce *CSPG4*-expressing pluripotent precursors or an obscure differentiated epithelial lineage, both distinct from the adult ductal cells and giving rise to certain neoplasms. *CSPG4*-expressing PDAC variants are of anaplastic origin (HS766T) or have strong mesenchymal features (Panc1) [56]. These cells might share a common early pancreatic precursor, which might even be the same cell as for SCA but with a different mutation, permitting or facilitating a malignant transformation.

Nonetheless, even knowing which marker should be used, the search for a normal precursor will be a challenge. The shedding of *CSPG4*, the abundance of circulating isoform and its ability to bind surface-retained extracellular molecules might impede exact identification of the *CSPG4*-producing precursor. The level of constitutive mRNA expression might be insufficient for in situ detection, although it might become readily detectable once cells are propagating and/or a gene is up-regulated and ‘fixed’ by a hypoxia-mimicking genetic aberration. As an alternative for the derivation of a normal counterpart, one could attempt human islets, shown to contain a distinct population of mesenchymal stem cells able to form *CSPG4*-positive clusters not of ductal, endothelial, or hematopoietic origin, which however, co-express mesenchymal and pancreatic endocrine markers [10]. Another way might be to establish in vitro systems reproducing the process of pancreatic differentiation [57]. For example, culturing mouse embryonic stem cells on a monolayer of mesonephros-derived cell line M15 replicates a multistep divergence of the ectoderm, mesendoderm and definitive endoderm, all the way to *PDX1*-expressing progenitors. The latter have been found to mature to all pancreatic lineages (endocrine, exocrine and ductal) upon engraftment under the kidney capsule of SCID mice [58].

While exploring the SCA–hypoxia link, we could not ignore the fact that only 22–57% of SCA cases are associated with VHL syndrome and somatic inactivation of the *pVHL* gene at chromosome 3p [59], [60]. In 50% of sporadic SCA, tumors have shown allelic loss of a putative tumor suppressor on chromosome 10q [60]. Our screening for hypoxia pathway-related genes identified the *HIF1a*-inhibitor *HIF1AN* at the 10q24 locus. Preliminary qRT-PCR measurements revealed a significant reduction of this gene’s mRNA expression in SCA biopsies compared to other pancreatic entities (unpublished observation). Recently, pancreatic deletion of the *LKB1/*S*TK11* gene in mice has been shown to result in acinar polarity defects and serous cystic neoplasms [35]. *LKB1* mutation also affects hypoxic signaling [36], [37]. In humans, *LKB1* loss-of-function mutations have been linked to the development of Peutz-Jeghers syndrome and IPMNs, both high-risk factors for pancreatic cancer [30], [61], [62]. In addition to the implication of candidate mutations for SCA, these data may link the well-established cell/stratum regulatory activity of *CSPG4* to cell polarity disorders. It is most likely that (membranous) *CSPG4* marks definite epithelial lineage, different from that of an adult pancreatic cell and prone to cyst formation upon ‘pseudo-hypoxic’ *CSPG4* overexpression in association with transforming p*VHL, HIF1AN* and *LKB1* mutations.

Altogether, our data indicate that *CSPG4* does not contribute to pancreatic carcinogenesis but might be of relevance for cell polarity disorders. *CSPG4* emerged as lineage marker, gaining hypoxic sensitivity upon cellular transformation. Surficial retention of *CSPG4* on expanding tumor cells improves its standing as a potential therapeutic target [63], [64]. As our study is the first to evaluate circulating s*CSPG4*, we cannot yet relate our findings to other diseases. The diagnostic relevance of the ‘drop and restoration’ pattern and pancreatic isoforms being clearly distinct (from melanoma-specific antigen), or just unsheddable (in contrast to cervical carcinoma-specific antigens), remains to be explored.
